# Structure and Evolution of Mediterranean Forest Research: A Science Mapping Approach

**DOI:** 10.1371/journal.pone.0155016

**Published:** 2016-05-09

**Authors:** Pierfrancesco Nardi, Giovanni Di Matteo, Marc Palahi, Giuseppe Scarascia Mugnozza

**Affiliations:** 1 Research Unit for Climatology and Meteorology applied to Agriculture, Consiglio per la ricerca in agricoltura e l’analisi dell’economia agraria, Rome, Italy; 2 European Forest Institute, Joensuu, Finland; 3 Department for Innovation in Biological, Agro-food and Forest Systems, University of Tuscia, Viterbo, Italy; Center for International Forestry Research (CIFOR), INDONESIA

## Abstract

This study aims at conducting the first science mapping analysis of the Mediterranean forest research in order to elucidate its research structure and evolution. We applied a science mapping approach based on co-term and citation analyses to a set of scientific publications retrieved from the Elsevier’s Scopus database over the period 1980–2014. The Scopus search retrieved 2,698 research papers and reviews published by 159 peer-reviewed journals. The total number of publications was around 1% (N = 17) during the period 1980–1989 and they reached 3% (N = 69) in the time slice 1990–1994. Since 1995, the number of publications increased exponentially, thus reaching 55% (N = 1,476) during the period 2010–2014. Within the thirty-four years considered, the retrieved publications were published by 88 countries. Among them, Spain was the most productive country, publishing 44% (N = 1,178) of total publications followed by Italy (18%, N = 482) and France (12%, N = 336). These countries also host the ten most productive scientific institutions in terms of number of publications in Mediterranean forest subjects. *Forest Ecology and Management* and *Annals of Forest Science* were the most active journals in publishing research in Mediterranean forest. During the period 1980–1994, the research topics were poorly characterized, but they become better defined during the time slice 1995–1999. Since 2000s, the clusters become well defined by research topics. Current status of Mediterranean forest research (20092014) was represented by four clusters, in which different research topics such as biodiversity and conservation, land-use and degradation, climate change effects on ecophysiological responses and soil were identified. Basic research in Mediterranean forest ecosystems is mainly conducted by ecophysiological research. Applied research was mainly represented by land-use and degradation, biodiversity and conservation and fire research topics. The citation analyses revealed highly cited terms in the Mediterranean forest research as they were represented by fire, biodiversity, carbon sequestration, climate change and global warming. Finally, our analysis also revealed the multidisciplinary role of climate change research. This study provides a first holistic view of the Mediterranean forest research that could be useful for researchers and policy makers as they may evaluate and analyze its historical evolution, as well as its structure and scientific production. We concluded that Mediterranean forest research represents an active scientific field.

## Introduction

Forest research in the Mediterranean region has been traditionally seen as handicapped by its fragmentation, its limited means, and occasional outdating and isolation. In addition, the low benefits that Mediterranean forests provide to forest-based industries—compared to other European forests-have made it difficult to attract interest and funds from the private sector. For this reason, specific European initiatives, like a specific Mediterranean forest research ERA-Net scheme- FORESTERRA or the European Forest Institute Mediterranean Regional Office have focussed during the last years in developing new ways to overcome this situation through new research partnerships, networking, capacity building, and information sharing between forest research organizations.

Mediterranean forest ecosystems provide multiple goods and services that are crucial to the socioeconomic development of the Mediterranean region’s rural areas as well as to the welfare of its urban populations. Advancing scientific knowledge and fostering innovation is essential, then, to ensure the sustainable management of Mediterranean forests and to build a knowledge-based bio-economy in the region. The countries of the Mediterranean basin, as well as those of other Mediterranean Climate Areas, face similar challenges regarding the sustainability of forest ecosystems and the delivery of crucial goods and services that they provide in a context of rapid global changes. Therefore, it is of critical importance to reinforce scientific cooperation on Mediterranean forests through transnational EU-Mediterranean and transcontinental cooperation among Mediterranean Climate Areas (California, Australia, South Africa, Chile) in order to reduce fragmentation and maximise the impact of research activities.

Despite the fact that research on Mediterranean forest ecosystems has received much attention during recent decades, there have been few attempts at evaluating Mediterranean forest research using bibliometric/scientometric studies. For example, traditional bibliometric methods have been applied to analyze publication trends related to fire regimes, water availability, biosphere-atmosphere interactions and ecological diversity in Mediterranean terrestrial ecosystems [1]. Further studies using bibliometric approaches have been applied to Forest Ecology [2] and to forest journals [3, 4]. However, more advanced bibliometric approaches known as science mapping or bibliometric mapping have been used to derive new insights by identifying trends, or clusters, in the bibliographic data sets associated with a field of study [5]. Specifically, science mapping is a spatial representation of how disciplines, fields, specialties, documents and authors are related to one another [6] and aims at displaying the structural and dynamic aspects of scientific research. Depending on the specific aim of the study, such mapping would show relations among different units of analysis (i.e., authors, documents, journals, and keywords) and are usually constructed based on citation, co-citation, bibliographic coupling data and co-occurrences of keywords. This approach has been applied to several research fields such as biodiversity [7], remote sensing [8], climate engineering [9], renewable energies [10], and carbon storage [11]. However, to our knowledge, the science mapping approach has not been used previously to analyze the research in Mediterranean forest ecosystems. Considering that many studies carried out in Mediterranean forest ecosystems have already been published, a bibliometric mapping analysis of this literature could serve as an alternative and innovative way for revealing the evolution and the structure of the research in Mediterranean forest ecosystems. We used, therefore, an advanced visualization methodology based on co-term analysis to identify clusters of related terms.

Additionally, a citation analysis was performed to reveal the citation impact of terms displayed into the maps. More specifically, we aimed at: (i) identifying trends in scientific publication outputs, and number of publications published by journals, countries and research institutions; (ii) providing a general field overview by visualizing main research areas, their relations and how they developed over time; (iii) identifying the most cited terms.

To accomplish these goals, a set of scientific publications related to Mediterranean forests were retrieved via Elsevier’s Scopus database. This study will produce visual representations of the research carried out in Mediterranean forest ecosystems and therefore will provide useful tools for the scientific community engaged in Mediterranean forest research to realize their advancements in the field. This research will assist policy makers when they have to set forest policies and make strategic decisions. Such an approach could serve as an alternative and innovative way for revealing global research trends in Mediterranean forest ecosystems.

## Materials and Methods

### Data gathering

Scopus database was used to retrieve bibliographic records related to Mediterranean forest research for the period 1980–2014. We did not use Web of Science because it was not available to us, whereas Google Scholar offers results of inconsistent accuracy [12, 13]. Scopus search was limited to papers and reviews written in English. To identify relevant Mediterranean forest publications, we used the following keywords in the combined field of title, abstract and keywords:

mediterranean PRE/1 forest* OR mediterranean PRE/1 forest* AND nitrogen OR mediterranean PRE/1 forest* AND carbon OR mediterranean AND "forest* management" OR mediterranean AND silviculture OR mediterranean AND agroforestry OR mediterranean AND "urban forest*" OR mediterranean AND ecophysiology OR mediterranean AND "wood harvesting" OR mediterranean AND "land use" OR mediterranean AND "landscape ecology" OR mediterranean PRE/1 forest* AND "aboveground biomass" OR mediterranean PRE/1 forest* AND climate OR mediterranean PRE/1 forest* AND fire OR mediterranean PRE/1 forest* AND "atmospher* change" OR mediterranean PRE/1 forest* AND emissions OR mediterranean PRE/1 forest* AND "trace gases" OR mediterranean PRE/1 forest* AND "nitro* oxides" OR mediterranean PRE/1 forest* AND ozone OR mediterranean PRE/1 forest* AND water OR mediterranean PRE/1 forest* AND diversity OR mediterranean PRE/1 forest* AND "ecosystem processes" OR mediterranean PRE/1 forest* AND "ecosystem function*” OR mediterranean PRE/1 forest* AND "forest genetics" OR mediterranean PRE/1 forest* AND "forest tree breeding" OR mediterranean PRE/1 forest* AND "forest biotechnology" OR mediterranean PRE/1 forest*AND bioeconomy AND NOT mediterranean PRE/1 sea AND NOT marine sea.

Some limitations of our searching strategy should be mentioned. First, the search was restricted to publications written in English. Second, as the data set is based on articles and reviews, we may have missed information contained in other bibliographic sources such as books and proceedings. Third, data were collected using only the Scopus database.

### Bibliometric mapping and clustering

Based on retrieved publications we constructed a number of bibliometric maps using the VOSviewer software (freely available at www.vosviewer.com), specifically developed for creating, visualizing and exploring science’s bibliometric maps [14]. We produced the so-called term maps, also referred as co-word maps [15]. A term map is a two-dimensional representation of a field, in which strongly related terms are located close to each other and less strongly related terms are located further away from each other. Thus, term maps provide overviews for identifying the structure of a field. Thanks to natural language processing techniques employed by the software, all terms occurring in titles and abstracts of publications were analyzed. A linguistic filter is then applied to filter out not relevant terms as well as terms occurring in a small number of publications [16]. To display the elements on maps, the software uses the VOS mapping technique [14]. The idea of VOS mapping technique is to minimize a weighted sum of squared Euclidean distances between all pairs of items through an optimization process. This mapping approach allows lying out terms on the map in a way that the distance between each pair of terms (i.e., *i* and *j*) represents their similarity as accurately as possible. Specifically, similarities among terms (*AS*_ij_) are calculated based on their number of co-occurrences in the title or abstract of the same publication according to the following equation:

$$AS_{ij}=\;\frac{c_{ij}}{c_{i}\;c_{j}}$$

where c_ij_ is the number of publications in which two terms *i* and *j* occur together and c_*i*_ and c_*j*_ represent the number of publications in which each one appears. The larger the number of publications in which two terms co-occur, the stronger the terms are considered to be related to each other. Therefore, terms that often co-occur in the same publications are located close to each other in a term map and less strongly related terms (low co-occurrence) are located further away from each other. Each term is represented by a circle, where its diameter and the size of its label indicate the number of publications that have the corresponding term in their title or abstract. To identify clusters of related terms, the software uses a weighted and parameterized variant of modularity-based clustering, that is the VOS clustering technique [17, 18]. A cluster can be understood as a research theme in which one or more research topics can be identified. Hereafter, we will refer to maps displaying clusters as term maps.

We also produced term citation maps in which the color of a term is determined by the average citation impact of the publications in which the term occurs. Specifically, in term citation maps, colors reflect the average citation impact for the term rather than by cluster.

To avoid bias related to the age of a publication, the number of citations it received is divided by the average number of citations of all publications appearing in the same year. This produces a publication’s normalized citation score range from 0 to 2, where the colors are assigned according to these scores. The colors ranging from blue (average score of 0) to green (average score of 1) to red (average score of 2 or higher). Therefore, a blue (cold) or red (hot) term indicates that publications in which the term occurs have low and high average citation impacts respectively [19]. To avoid overlapping labels, only a subset of all labels is displayed in the maps. However, all maps reported in this paper can be freely explored by using the VOSviewer text files we provided (to do this, please see Supporting Information: S1 and S2–S6 Figs).

In addition, a thesaurus file (text file) for VOSviewer was created to ensure consistency for different spelling and synonyms (an example: Leaf area index is changed with LAI). Before obtaining maps, VOSviewer offers the possibility to clean out the data by omitting those terms considered not relevant for analyses. We performed, therefore, the cleaning by omitting terms related to time, publishers’ names and geographical locations (i.e., names of cities). It should be noted however, that a term maps represents a simplified version of reality and this can lead to loss of information and to a partial representation of the investigated field. This limitation should be taken into account when interpreting a term map.

Splitting data into different periods is a common approach to analyze the evolution of a scientific field [20]. Therefore, the retrieved bibliographic data were split into the following six periods: 1980–1989, 1990–1994, 1995–1999, 2000–2004, 2005–2009 and 2010–2014. For each period, term maps as well as term citation maps were constructed. Due to the low number of articles published during the first ten years of the survey, this period was collapsed into a single time slice (i.e., 1980–1989).

## Results

### Publication trends: countries and research institutions

**Fig 1:**
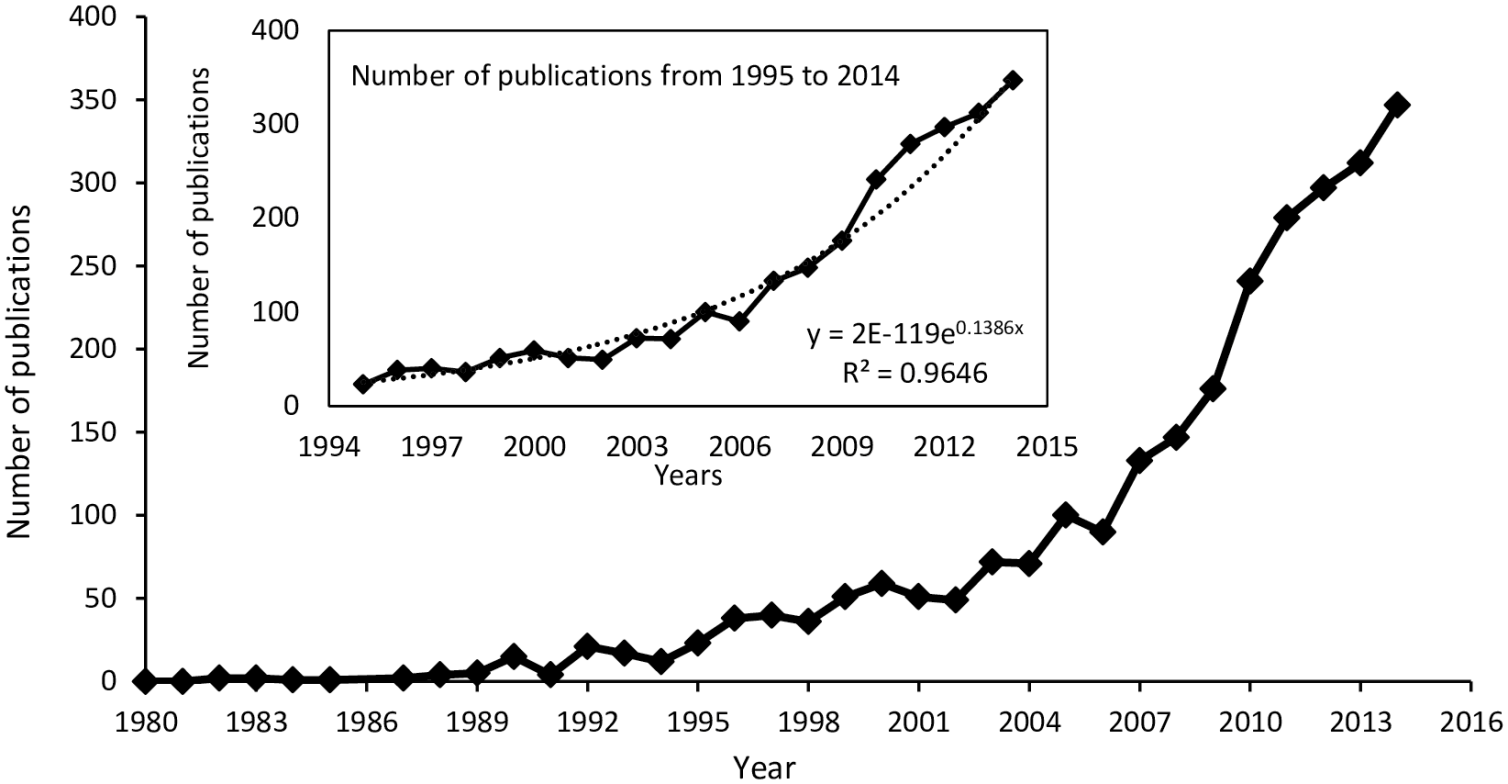
Trends in Mediterranean forest publications from 1980 to 2014. The boxed graph shows the exponential growth (dotted line) of publications during the time slice 1995–2014.

We retrieved a total of 2,698 scientific publications from Scopus database over 34 years. Approximately, 97% of them were research papers while review papers represented the remaining 3%. Fig 1, depicts the Mediterranean forest publication frequencies from 1980 to 2014. During the period 1980–1989, the total number of publications was only around 1% (N = 17) and they reached 3% (N = 69) in time slice 1990–1994. The number of publications increased exponentially during 1995–2014, with more than 50% of them published during the last five years.

Altogether, 159 journals have been involved in the field. Table 1 lists the 33 journals publishing at least 20 publications from 1980 to 2014. The most active journals were *Forest Ecology and Management* (N = 153, 5.67%), *Annals of Forest Science* (N = 46, 1.70%), *Catena* (N = 44, 1.63%), *Biodiversity and Conservation* (N = 40, 1.48%) and *Agriculture*, *Ecosystems & Environment* (N = 39, 1.45%).

**Table 1:** Most active journals in Mediterranean forests research from 1980 to 2014.

Journals	Publications	% of total
Forest Ecology and Management	153	5.67
Annals of Forest Science	46	1.70
Catena	44	1.63
Biodiversity and Conservation	40	1.48
Agriculture, Ecosystems & Environment	39	1.45
European Journal of Forest Research	39	1.45
Landscape and Urban Planning	38	1.41
Plant Ecology	34	1.26
Agroforestry Systems	33	1.22
International Journal of Wildland Fire	32	1.19
Plos One	31	1.15
Biological Conservation	30	1.11
Science of the Total Environment	29	1.07
Landscape Ecology	29	1.07
Environmental Management	28	1.04
Acta Oecologica	28	1.04
Journal of Hydrology	28	1.04
Journal of Vegetation Science	28	1.04
Land Degradation and Development	27	1.00
Global Change Biology	25	0.93
Journal of Environmental Management	24	0.89
International Journal of Remote Sensing	24	0.89
Geomorphology	24	0.89
Plant Biosystems	23	0.85
Ecological Modelling	22	0.82
Plant and Soil	22	0.82
Environmental Monitoring and Assessment	22	0.82
Geoderma	21	0.78
Remote Sensing of Environment	21	0.78
Ecological Indicators	21	0.78
Tree Physiology	21	0.78
Forest Systems	21	0.78
Soil Biology and Biochemistry	20	0.74

**Fig 2:**
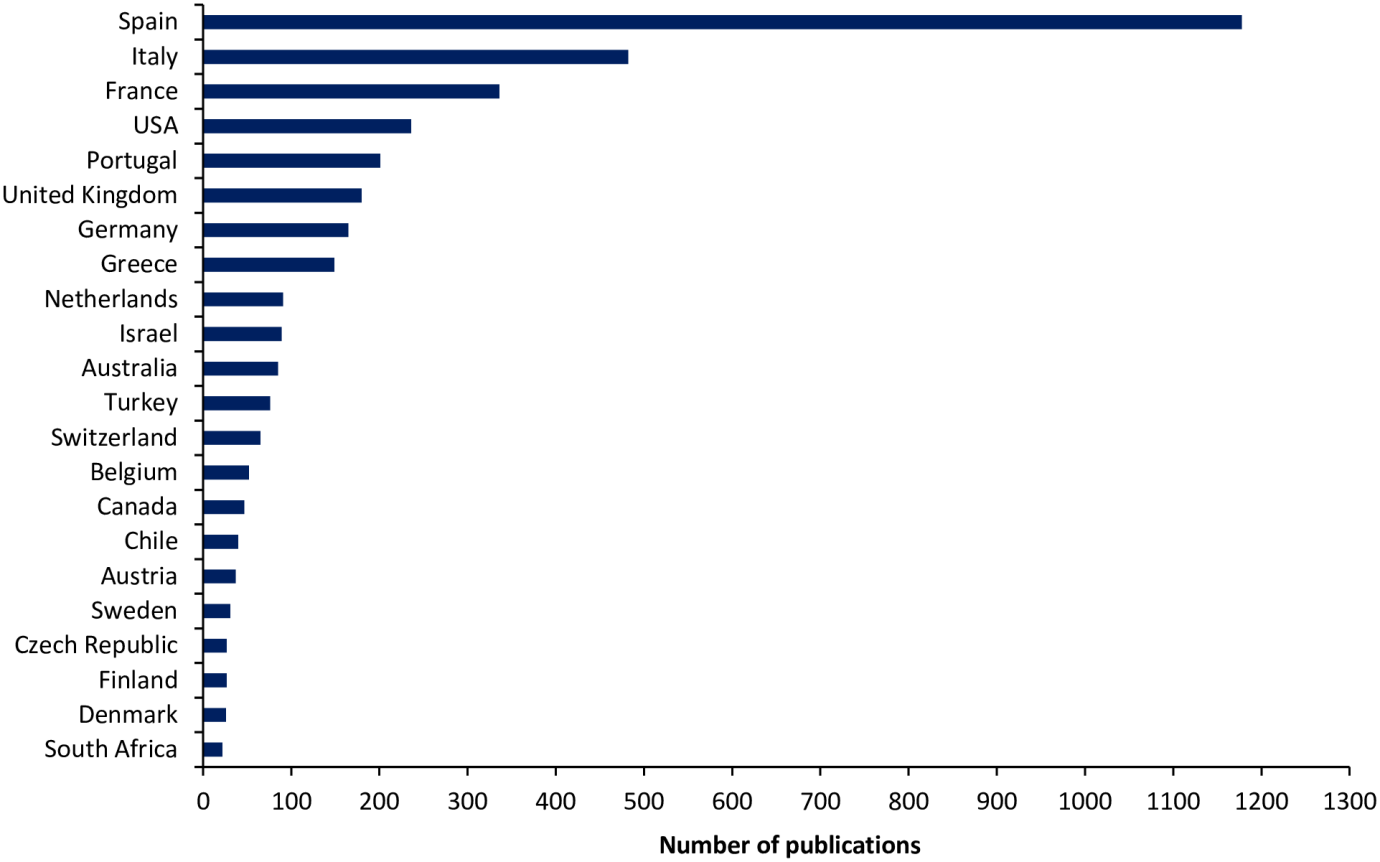
Countries publishing at least 20 papers related to Mediterranean forest research from 1980 to 2014.

There were 88 countries publishing at least one publication related to Mediterranean forest research. Among them, Spain published 44% of total publications (N = 1,178), followed by Italy (18%, N = 482) and France (12%, N = 336) (Fig 2). These countries also host the ten most productive scientific institutions in publishing Mediterranean forest research (Table 2).

**Table 2:** The ten most productive institutions publishing articles or reviews on Mediterranean forests from 1980 to 2014.

Affiliations	Publications	% of total	Country
Universitat de Barcelona	112	4	Spain
Centre de Recerca Ecològica i Aplicacions Forestals	106	4	Spain
Consiglio Nazionale delle Ricerche	84	3	Italy
Universidad Autonoma de Barcelona	70	3	Spain
Instituto Nacional de Investigacion y Tecnologia Agraria y Alimentaria	69	3	Spain
Centre National de la Recherche Scientifique	68	3	France
Centre d'Ecologie Fonctionnelle et Evolutive	62	2	France
Università degli Studi della Tuscia Viterbo	61	2	Italy
Universidad Politecnica de Madrid	55	2	Spain
Universidad de Castilla-La Mancha	50	2	Spain

### Evolution of research topics and their citation impacts

Figs 3–12 display the term maps and term citation maps across five periods since the 1990s. We should note that in term maps colors are used to identify clusters of related terms, while in citation maps the colors indicate the average citation impact of publications in which the term occurs.

**Fig 3:**
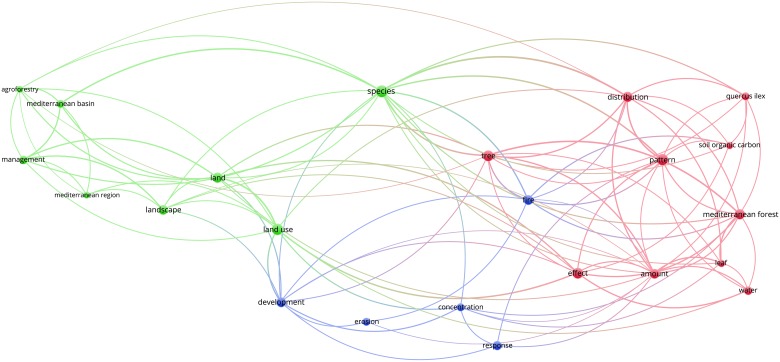
Term map based on Mediterranean forest publications from the time slice 1990–1994. Lines (100) indicate co-occurrence links between terms.

**Fig 4:**
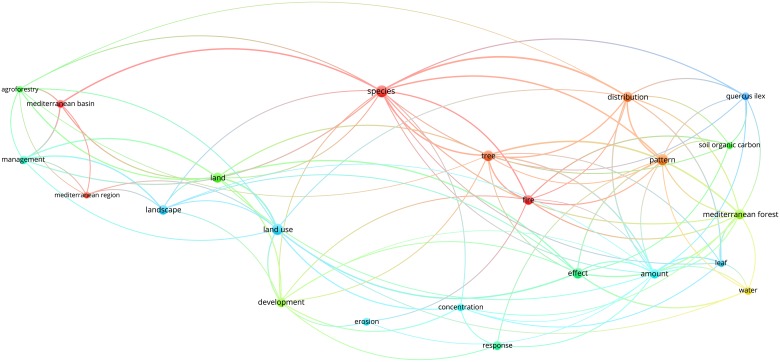
Term citation map based on Mediterranean forest publications from the time slice 1990–1994. Lines (100) indicate co-occurrence links between terms.

**Fig 5:**
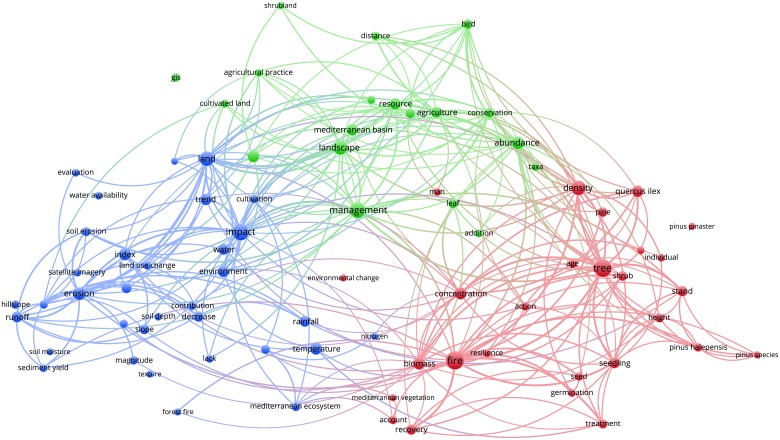
Term map based on Mediterranean forest publications from the time slice 1995–1999. Lines (300) indicate co-occurrence links between terms.

**Fig 6:**
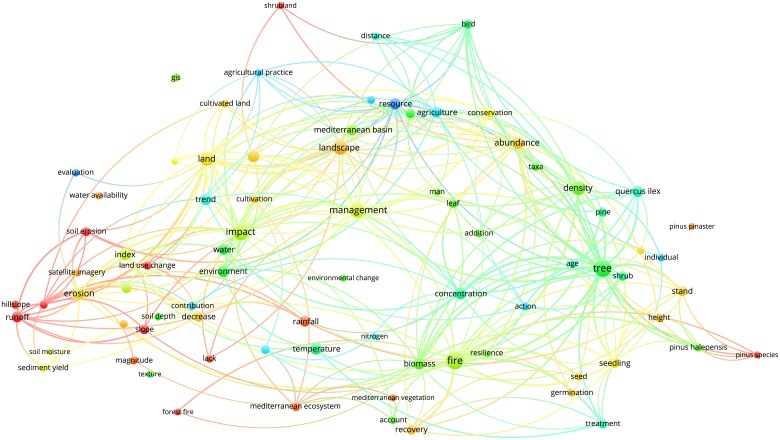
Term citation map based on Mediterranean forest publications from the time slice 1995–1999. Lines (300) indicate co-occurrence links between terms.

**Fig 7:**
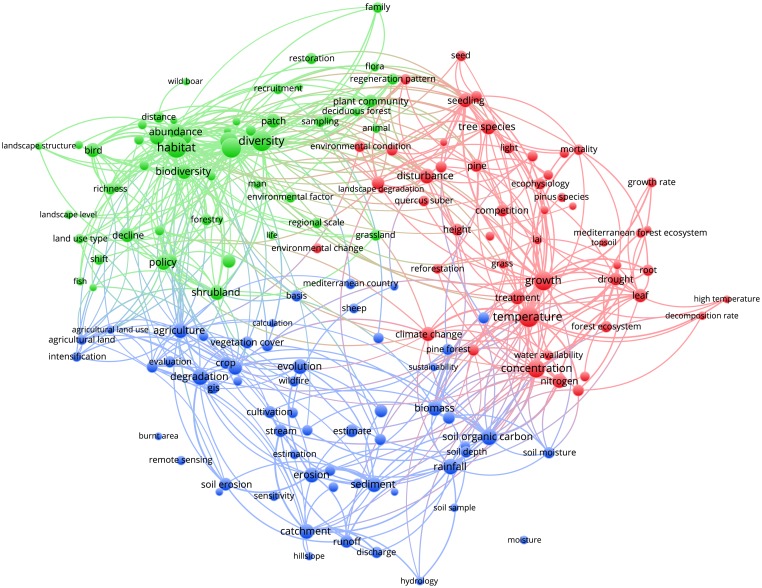
Term map based on Mediterranean forest publications from the time slice 2000–2004. Lines (500) indicate co-occurrence links between terms.

**Fig 8:**
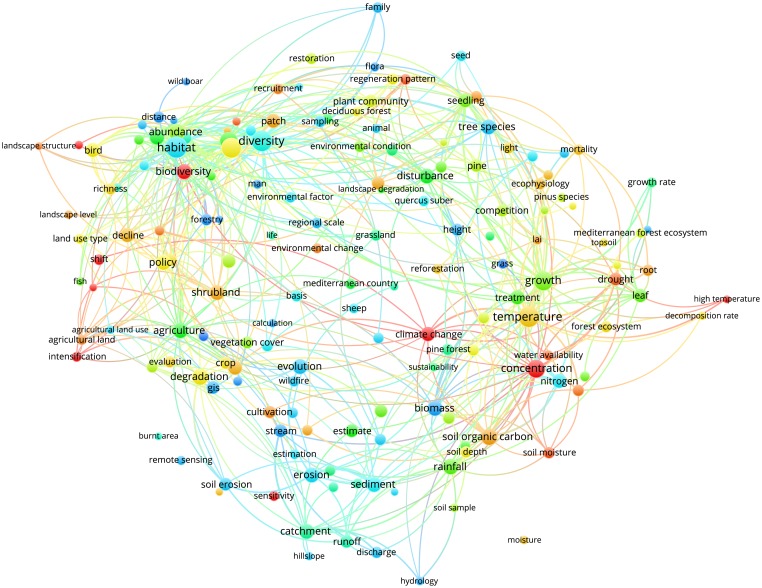
Term citation map based on Mediterranean forest publications from the time slice 2000–2004. Lines (500) indicate co-occurrence links between terms.

**Fig 9:**
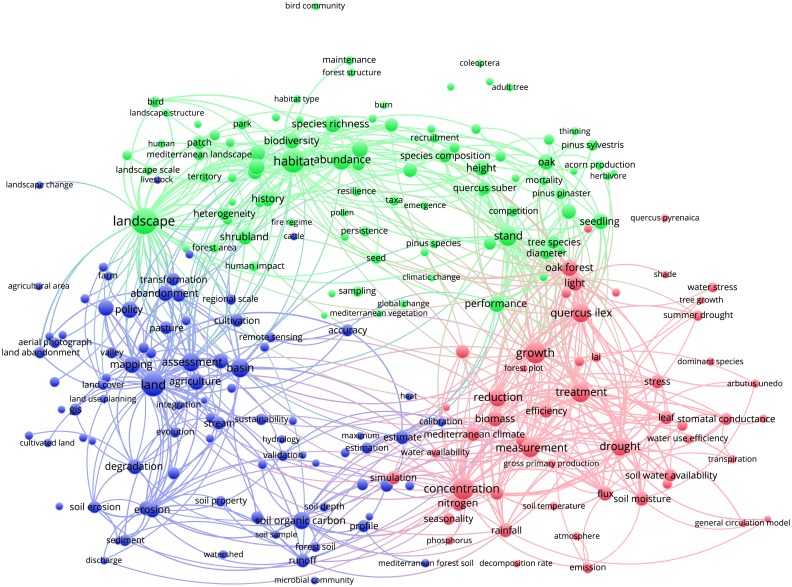
Term map based on Mediterranean forest publications from the time slice 2005–2009. Lines (600) indicate co-occurrence links between terms.

**Fig 10:**
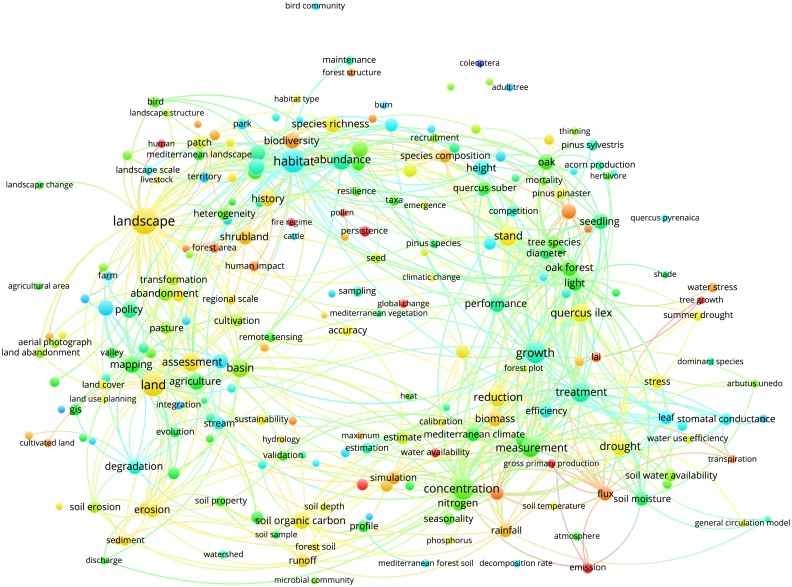
Term citation map based on Mediterranean forest publications from the time slice 2005–2009. Lines (600) indicate co-occurrence links between terms.

**Fig 11:**
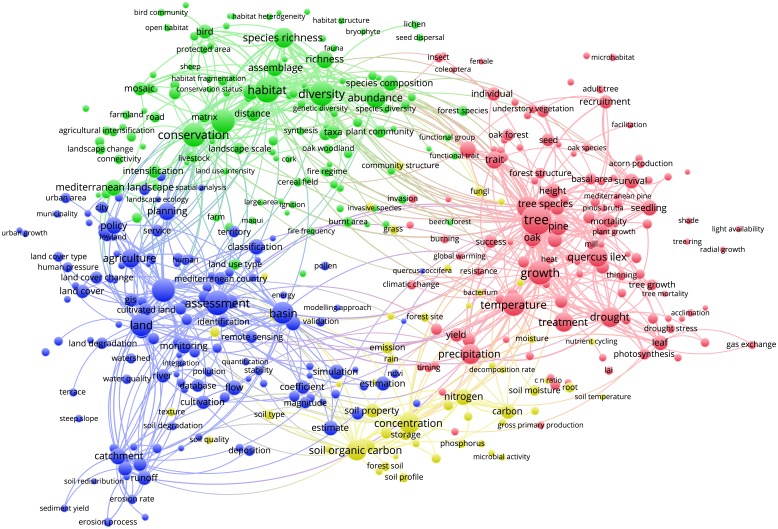
Term map based on Mediterranean forest publications from the time slice 2010–2014. Lines (600) indicate co-occurrence links between terms.

**Fig 12:**
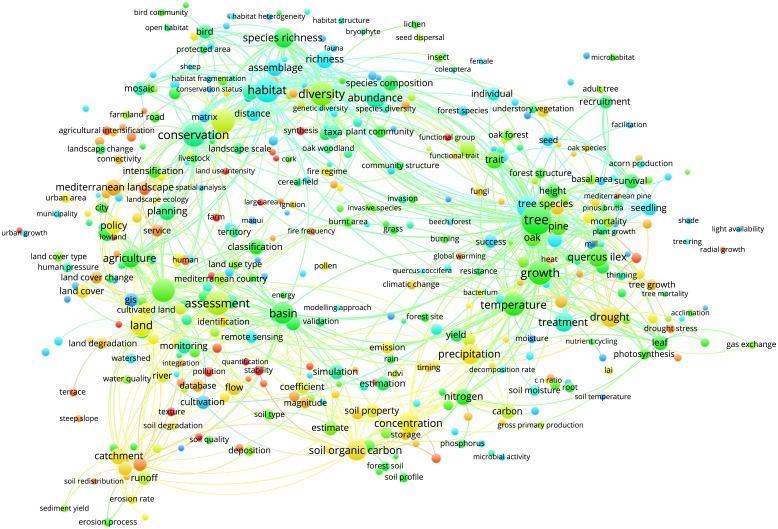
Term citation map based on Mediterranean forest publications from the time slice 2010–2014. Lines (600) indicate co-occurrence links between terms.

For the time slice 1980–1989, the term maps (not shown) contained only terms regarding “Mediterranean forest”, “fire” and “vegetation”. Terms citation analysis revealed that publications reporting terms like “vegetation” were cited more frequently compared to those reporting “fire” or “Mediterranean forest”.

Fig 3 shows the term map constructed for the period 1990–1994. The 23 terms displayed on the map are grouped in three clusters (blue, red and green). Green cluster (left side) grouped terms such as “landscape”, “land-use”, “agroforestry”, “management”, “land” and “species”. Terms such as “distribution”, “tree”, “*Quercus ilex”* and “water” appeared in the red cluster (right side), while terms such as “erosion” and “fire” are grouped in the blue cluster at the bottom side of the map.

The citation map revealed highly cited terms such as “species”, “Mediterranean region”, “Mediterranean basin” and “fire”, i.e. upper side of the map (Fig 4).

The term map for the period 1995–1999 was constructed on 80 terms, which were grouped in three clusters (blue, red and green) (Fig 5). Green cluster (upper side of the map) grouped terms roughly associated to the landscape and land-use research topics (i.e., management, landscape, conservation and cultivated land). Red cluster (right side of the map) grouped terms related to fire and forest regeneration research topics (i.e., fire, seedling, germination and seed). Interestingly, this cluster also showed emerging terms such as “environmental change” and “resilience”. Blue cluster (left side of the map) grouped terms related to the research topic of soil erosion (i.e., erosion, hillslope, runoff, rainfall and land-use change).

Looking at the citation map (Fig 6), we should note that hot terms like “runoff”, “soil erosion”, “land-use change”, “forest fire” and “hillslope” are located in the left side of the map and fall in the blue cluster.

For the time slice 2000–2004, the number of terms displayed on the term map increased to 162, but as for previous periods, they were grouped into three clusters (Fig 7).

Green cluster (left side of the map) contained terms such as “biodiversity”, “conservation”, “species richness”, “diversity”, “composition”, “landscape structure”, “landscape level”, “landscape change” and “land-use history”, mainly related to landscape and biodiversity conservation research topics. This cluster also revealed terms related to forest restoration/regeneration research topic, i.e. “restoration”, “recruitment”, and “regeneration pattern”, as well as novel terms such as “policy”, and “wild boar”. During this period, a novel ecophysiology research topic emerges into the red cluster (right side of the map) as it showed terms related to the effect of climate change (i.e., climate change, temperature, high temperature, environmental change, decomposition rate and water availability) on plant ecophysiology (i.e., ecophysiology, LAI, light and photosynthesis). However, additional terms related to forest restoration/regeneration research such as “seedling”, “seed”, “survival” and “tree species”, also occurred in the upper side of this cluster. Blue cluster grouped terms related to land-use and degradation (i.e., agriculture, agricultural land-use, degradation and intensification), soil erosion and water management (i.e., catchment, erosion, soil erosion and hydrology) and soil (i.e., soil moisture, soil property and soil organic carbon) research topics.

The citation map displayed several terms with above average citation impact (i.e., biodiversity, environmental change, climate change, drought and high temperature) (Fig 8).

Fig 9 shows the term map for the time slice 2005–2009 based on 234 terms, which were grouped into three clusters. As for the previous period, the green cluster (on the top side of the map) still groups terms related to the biodiversity, forest management, conservation and forest restoration. Yet, we should note the great occurrence of term “landscape” and novel terms related to fire’s research like “fire regime”. This cluster also accommodates terms such as “climate change” and “climatic change”, previously located in the red cluster.

The red cluster becomes much more focused on ecophysiology and ecosystem functioning research and, more specifically, to tree-atmosphere interactions (i.e., atmosphere, flux, emission, gross primary production, LAI, stomatal conductance and transpiration), even if terms related to the silviculture and tree biometrics research (i.e., tree growth, growth, and biomass) have occurred. The blue cluster (on the left part of the map) becomes bigger compared to the previous period and accommodates terms generally related to land-use and degradation, policy and land management (i.e., policy, land abandonment, GIS and aerial photograph), erosion and water management (i.e., basin, stream, river and erosion) and soil (i.e., soil organic carbon, microbial community, and forest soil) research topics.

By looking the term citation map (Fig 10), we should note that hot terms occurred mainly in the upper side of the map (i.e., human, human impact, fire regime and global change) as well as in the bottom-right side (i.e., water availability, LAI, gross primary production, transpiration, flux and emission). Yet, a term such as “prediction”, which acts as bridge between red and blue clusters (bottom side of the map), represents a hot term.

The term map for the last period (i.e., 2010–2014) was constructed based on 453 terms (Fig 11). This map was characterized by four clusters since terms related to soil research, previously located into the blue cluster, appeared as a separate yellow cluster (bottom side of the map).

The green cluster grouped terms related to three research topics such as biodiversity and conservation (i.e., biodiversity, conservation, biodiversity conservation, habitat, diversity and species richness), fire (i.e., fire regime, fire frequency, large fire, burnt area, ignition, bare soil, fire hazard and wildfire) and landscape (i.e., landscape, structure, and Mediterranean landscape). Additionally, this cluster has shared with the red cluster some terms related to the fauna research (i.e., bird, fauna, insect, coleoptera and female). The red cluster confirmed the presence of terms related to ecophysiology but it accommodates also some terms from silviculture and tree biometrics research. Therefore, ecophysiological research was still well represented by the red cluster and mainly focused on the effects of climate change on plant responses and ecosystem functioning (i.e., temperature, precipitation, drought, treatment, photosynthesis, stomatal conductance, transpiration, acclimation, and gross primary production). However, novel terms more specifically related to silviculture, tree biometrics and dendrochronology research such as “basal area”, “radial growth”, “yield”, “tree rings”, “forest structure”, “stand”, “height”, “diameter”, “survival”, “mortality”, “stem”, “understory vegetation”, and “thinning” have occurred, thus highlighting the importance of the silvicultural practices in mitigating the negative effect of climate change (i.e., research linked to the adaptive management). This cluster also showed research topics related to forest regeneration/restoration (i.e., natural regeneration, regeneration pattern, and recruitment), whereas it was previously located in the green cluster.

As previously stated, terms such as “soil organic carbon”, “carbon sequestration”, “microbial structure”, “CO_2_”, “emission” as well as terms related to plant nutrients (i.e., nitrogen and phosphorus) and soil properties, are grouped into a new yellow cluster, thus representing the research topic of soil. This cluster also accommodates terms related to the carbon sequestration at the forest scale such as “carbon sequestration”, “carbon”, “CO_2_”, “plant cover”, “forest site”, indicating increasing interrelations between soil and tree carbon research. Different research topics can be identified looking at the blue cluster. Starting from the bottom side of the map and proceeding clockwise, this cluster showed terms such as “catchment”, “runoff”, “basin”, “erosion”, “erosion rate” and “erosion process” clearly related to the research topics of soil erosion and water management. The terms such as “land cover change”, “cultivated land”, “agricultural practice” and “land” are related to the land-use change research topic. The terms related to forest policy and services such as “ecosystem services” “policy”, “service”, and “transformation” appeared in the closeness of green cluster. Finally, this cluster also revealed terms related to the human activity and/or pressure such as “city”, “urban growth”, “urban area” and “municipality”.

The citation map for this period showed that highly cited terms are generally located in the left side of the map and they were related to fire, human impact, service and water management research (i.e., fire regime, large area, fire frequency, Mediterranean city, farm, agricultural intensification, ecosystem services, pollution and soil redistribution). Additional highly cited terms were “functional diversity”, “functional trait” and “carbon sequestration” (Fig 12).

## Discussion

### Publication trends: countries and research institutions

According to our survey, during the time slice 1980–1989, there were only 17 English papers focusing on Mediterranean forests, but the earliest publication was indexed by Scopus in 1982. We recognize that the number of scientific publications we retrieved represents a proxy of the scientific activity in the field as our findings may be biased, to some extent, by the database and the keywords used [21]. For instance, it is not clear whether the low number of publications collected from the period 1980–1989 would reflect a low publication activity. If this is the case, the above period can be considered as the beginning stage of the Mediterranean forest research. However, an alternative explanation for the observed publication trend is that data are biased by the database used. That is, the publications indexed in Scopus for this period may partially represent the scientific activity in the field.

Since 1990, the number of publications increased and reached the maximum value in 2014. Similar trends were reported in other studies focusing on Mediterranean ecosystems [1], engineering research [9], global climate change [22] and network ecology [23]. Furthermore, a bibliometric study conducted on 60 journals indexed under the category of “Forestry” by Thomson Reuters, reported that the total number of publications was 2,298 in 1997 and 4,825 in 2012 [24]. Based on these data, the ratio of Mediterranean forest publications to total published in forestry, can be roughly estimated by 1% and 6% for 1997 and 2012 years respectively. Moreover, while the number of publications published yearly related to Forest Ecology issues increased at a stable state from 2002 to 2011 [2], studies on Mediterranean forests increased exponentially. This finding shows that the research on Mediterranean forests represents a vibrant research field within Forest Sciences.

The 2,698 papers analyzed in our study were published by 159 journals. Moreover, 1,067 (40%) of them were published in the top 33 journals. *Forest Ecology and Management* was the most productive journal in terms of number of publications and this finding was in agreement with other studies [24, 25]. *Annals of Forest Science* and *Catena* journals ranked second and third respectively. We should note that while the first two journals focus mainly on forest subjects, *Catena* is a soil science journal focusing on geoecology and landscape evolution. This finding highlights the multidisciplinary of the research carried out on Mediterranean forests. Furthermore, the research interest on Mediterranean forest ecosystems is shared globally as there were 88 countries contributing with at least one publication to our data set. The five worldwide areas affected by Mediterranean climate conditions (i.e., Australia, South Africa, Chile, California, and Mediterranean Basin) were well represented by top 20 countries publishing at least 20 papers during the period 1980–2014. Yet, Spain, Italy and France had the greatest contribution to the Mediterranean forest research. Other Mediterranean countries such as Portugal, Greece, Israel and Turkey appeared in the top 20 as well. A survey conducted on 13 Mediterranean countries during the 2010 and 2011, revealed that France, Spain and Italy, account alone for 80% of the total budget dedicated to forest research [24, 25]. Therefore, it is not surprising that these three countries, as well as their research institutions are the main contributors in Mediterranean forest research.

### Evolution of research topics and their citation impacts

Through the science mapping analysis, we obtained a set of maps displaying clusters of co-occurring terms in which we identified different research topics. On the other hand, terms were also colored from blue to red depending on their average citation impact. Additionally, as the mapping approach was conducted on different periods, we were also able to study the temporal evolution of the Mediterranean forest research during the last 34 years.

During the time slices 1980–1989 and 1990–1994 the low number of terms displayed on the map as well as their vagueness did not allow for grouping into defined research topics. However, research interest on “fire” and “landscape” was highlighted by the presence of these key-terms during the time slices considered. Since 1995s more specific terms appeared, leading to a better clustering definition as well as of research topics. Comparing the maps over the study periods allows for the observance of the appearance and disappearance of terms or term's repositioning among clusters, allowing important information regarding the dynamic of research topics addressed in a specific area of maps to be obtained. For instance, during the period 1995–1999, fire research was conducted in relation to forest regeneration, as these two research topics were located close into the red cluster. Looking to the next period (2000–2004), it becomes evident that not only does fire research almost disappear from the Mediterranean research literature, but the only terms related to this topic (i.e., burnt area and wildfire), appear in the blue cluster together with soil erosion and land degradation research topics.

Interestingly, during this period the biodiversity issue emerged as a clear research topic in Mediterranean forest research, focusing mainly on specific components of the biodiversity such as species diversity (i.e., richness and species richness), ecosystem (i.e., habitat) diversity, and its conservation. This finding is in agreement with other studies that identified conservation as a significant target in biodiversity research [7].

Yet, during time slices 2005–2009 and 2010–2014, fire research was mainly conducted in relation to biodiversity and conservation research topics (green cluster). Similar observations can be drawn by looking at the position of other important terms such as “environmental change”, “global change”, “climate change”, “climatic change” and “global warming”. Yet, the occurrence of novel terms over the years was mirrored by increasing cluster complexities. In general, as disciplines mature and expand their background knowledge, specialization becomes inevitable as the amount of information becomes too large for any individual researcher to master [26]. The occurrence of novel and more specific terms, the increasing of cluster complexities, and the possibility to identify among each cluster different research topics, suggests that research on Mediterranean forests followed the same trajectory.

However, particular attention should be paid to the map obtained for the most recent period (i.e., 2010–2014) as it represents the current status of the Mediterranean forest research. Biodiversity is one of the main characteristics of Mediterranean forest ecosystems that goes beyond the traditional forest products. Our findings showed that biodiversity and its conservation were well represented by the green cluster in the upper side of the map, thus demonstrating that it is an important pillar of the Mediterranean forest research. Future climate changes will be likely affected by further increases in mean temperature, frequency and severity of extreme droughts and hot extreme events [27, 28], therefore the understanding of plant responses to the changing environment represents an important research challenge for the Mediterranean forest ecosystems [29]. Ecophysiology represents an important tool to understand how plants will respond to the changing environmental conditions through the examination of mechanisms driving the functioning, distribution, abundance, and productivity of tree species [30]. Our analysis revealed that ecophysiology is a stable and a well defined research topic in the Mediterranean forest research.

The yellow cluster was related to soil research with an emphasis on carbon sequestration, nutrients, microbial activity and its structure and soil emissions. The closeness of soil research to ecophysiology research in the red cluster also highlights a special attention towards the understanding of soil-forest–atmosphere feedbacks. This is known as a research line and needs special attention [1].

Finally, research topics related to the anthropogenic pressure (i.e., municipality, city, urban growth, urban area and human pressure), land-use change and degradation as well as water management were well represented in the blue cluster. Of chief importance is to note that since the 2000s, the analysis of maps enables the detection of two types of research, i.e. applied and basic. The former, was mainly located in upper and left sides of maps and corresponds mainly to research topics grouped into the green and blue clusters (i.e., landscape, biodiversity conservation, fire research, soil erosion, water management and land degradation). Conversely, the more basic research was mainly related to ecophysiology research displayed by the red cluster. It is worth mentioning that due to the appearance in the period 2000–2004, the term “policy” was always located in the left part of the maps, thus showing areas of major influence of the forest policies. The visualization methodology applied in this paper enables the identify of not only the structure of the Mediterranean forest research in terms of clusters and research topics but also the changing of citation impacts over time. An important observation is that research topics tend to fall in different clusters according to the time slice considered. Additionally, more basic research related to the ecophysiology was highly cited during the periods 2000–2004 and 2005–2009, but had lower citation impact compared to other areas during the last period 2010–2014. However, our results also point out that both fire and climate change have been highly cited topics over time. Moreover, we should note that starting from 1996 up to 2014, maps assumed a circular form with some terms located in the proximity of the center, thus pointing to their multidisciplinary role in Mediterranean forest research. This was because most of them are related to the changing climate (i.e., environmental change, climate change, climatic change, global change and global warming) for which several concerns have been expressed in the literature [1, 29, 31, 32, 33, 34]. Interestingly, the term “global warming” appeared only in the most recent period (2010–2014), thus highlighting a rising interest towards global warming effects on Mediterranean forest ecosystems.

## Conclusions

In an increasingly complex world, the role of science becomes more important than ever in making policymakers understand the causes and interactions of different challenges and opportunities, as well as the consequences of policy options. Moreover, because of the complex character of many policy issues, science-based information need to be based on strong intersectorial and interdisciplinary engagement, combining natural and social sciences. To complicate things even further, nowadays most policy issues are of a transnational nature, and can only be addressed in a meaningful way through collaboration between countries. In such context, bibliometric mapping represent a powerful approach to get insights into the state and nature of fields or disciplines. It aims to produce maps of the relations between certain units of interest such as journals, authors, research institutions and terms. To our knowledge, the present study represents the first attempt to apply a science mapping approach for evaluating structure and development of the Mediterranean forest research.

Through the analysis of 2,698 publications published from 1980 to 2014, we concluded the following: (i) the number of scientific publications increased exponentially since 1995, (ii) *Forest Ecology and Management* was the most active journal in publishing Mediterranean forest research, (iii) Mediterranean forest research was mainly led by Spain, Italy and France, (iv) biodiversity and conservation, forest restoration, climate change, land degradation and soil represent well defined research topics, characterizing the current status of the Mediterranean forest research, (v) fire and climate change research generally received much more citations over time and (vi) the multidisciplinary role of global change research. The analysis presented in this paper represents the first step in analyzing the structure of Mediterranean forest research. Future studies may address different but related issues such as the analysis of collaborations among different actors (i.e., authors, organizations and countries) as well as the characterization of the research carried out in different geographic regions.

## Supporting Information

S1 Fig Instructions. Instructions are given on how to import into the VOSviewer software the text files we provided in S2–S6 Figs.
(PDF)

S2 Fig A and B. Term map based on Mediterranean forest publications from the time slice 1990–1994. Term citation map based on Mediterranean forest publications from the time slice 1990–1994. (RAR)

S3 Fig C and D. Term map based on Mediterranean forest publications from the time slice 1995–1999. Term citation map based on Mediterranean forest publications from the time slice 1995–1999. (RAR)

S4 Fig E and F. Term map based on Mediterranean forest publications from the time slice 2000–2004. Term citation map based on Mediterranean forest publications from the time slice 2000–2004. (RAR)

S5 Fig G and H. Term map based on Mediterranean forest publications from the time slice 2005–2009. Term citation map based on Mediterranean forest publications from the time slice 2005–2009. (RAR)

S6 Fig I and J. Term map based on Mediterranean forest publications from the time slice 2010–2014. Term citation map based on Mediterranean forest publications from the time slice 2010–2014. (RAR)
