# Supplementary material for: Structure and Evolution of Mediterranean Forest Research: A Science Mapping Approach
Source: PLoS One. 2016 May 9;11(5):e0155016. doi: 10.1371/journal.pone.0155016 (PMC4861273; doi:10.1371/journal.pone.0155016)
Supplement: S1 Fig — Instructions are given on how to import into the VOSviewer software the text files we provided in S2–S6 Figs. (PDF) [file pone.0155016.s001.pdf]

Supplementary Material

Pierfrancesco Nardi, Giovanni Di Matteo, Marc Palahi, Giuseppe Scarascia Mugnozza

Instructions on how to import text files and visualize maps in VOSviewer

The following instructions help you to visualize term and citation maps reported in the printed version of the paper using the software VOSviewer freely available at: www.vosviewer.com.

The five folders you see refer to the following periods:

<folder> 90 94: period 1990-1994
<folder> 95 99: period 1995-1999
<folder> 00 04: period 2000-2004
<folder> 05 09: period 2005-2009
<folder> 10 14: period 2010-2014

Each folder contains three text files named Map, Network and Cluster colors that you need to import into VOSviewer to visualize the maps. To do this, please follow the steps listed below.

1) Download VOSviewer from the http://www.vosviewer.com/download and install it on your computer.

2) After the download is complete, double click to start the program.

3) Open the above folders and download, by right click, the text files on your computer.

4) Click on the “open” bottom in VOSviewer to import Map and Network text files into the software. Click OK to visualize the map (Fig. 1, Fig. 2a).

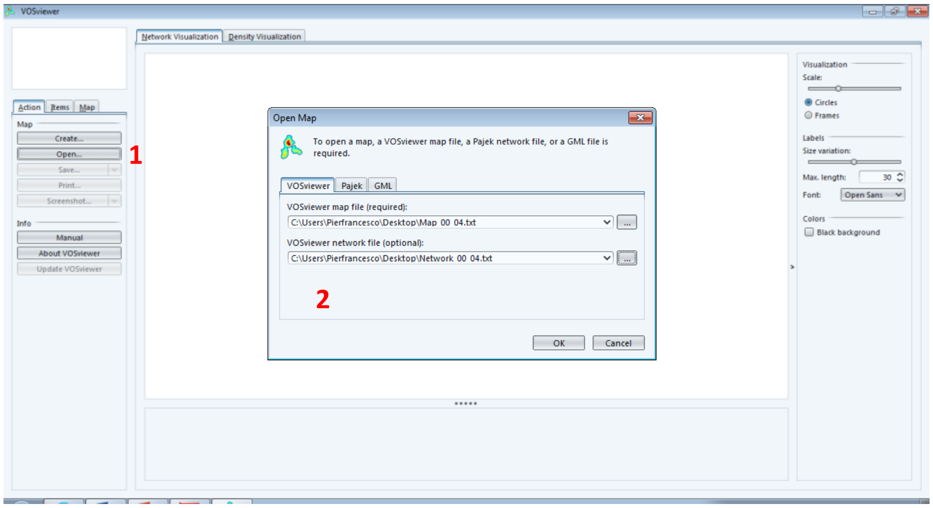

Figure 1. Example showing how to import and visualize a map in VOSviewer.

5) To visualize clusters with the same colors as those reported in the paper, the Cluster colors text file needs to be imported into the software. To this aim, click on the Cluster colors bottom (Fig. 2a) and select, by right click, import the Cluster color file using the new window as showed in figure 2b. This will allow you to visualize the map as reported in figure2c.

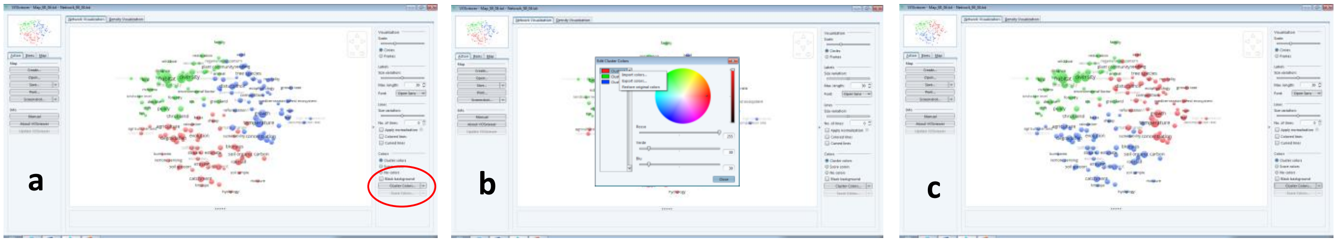

Figure 2. Importing cluster colors file into the software.

6) To visualize citation maps with the right score range, you need to click on the Score colors bottom and select the correct range. That is, Min. score equal to 0 and Max. score equal to 2.
